# Nursing the Sick

**Published:** 1881-06

**Authors:** 


### NURSING THE SICK.

*From "Hall's Family Doctor."*

GOOD nursing, a judicious attention to the sick and all the surroundings of a sick bed, would cure many cases of disease without the use of any medicine whatever; in other cases, where medicine cannot be borne, in consequence of the great debility of the patient, or the exhaustion of the system, it has been successful in restoring health and life for many years. And if families would heed the suggestions which follow, with reasonable fidelity, the attention of a physician could often be dispensed with, to say nothing of the comfort afforded to the sick and suffering, with all their discouragements and despondencies.

1. The sick-room should be up stairs if possible. There are certain pernicious emanations from the earth, besides the raw dampness and heaviness, which vitiate the air, and otherwise render it pernicious to the invalid. If there is no second story, the higher the bed from the floor the better. Pallets on the floor, cradles, lounges, cots and trundle-beds should be exchanged for high bedsteads.

2. By all means place the sick in the room in the building which faces the sun for the greater part of the day, although it may not have as many windows as others, and although it may be smaller, for the sunshine keeps it light and cheerful; it keeps it warm, a healthful warmth; it keeps it dry, and besides, imparts a life to the atmosphere breathed, which of itself is a very important element in bringing back vitality to an exhausted frame.

3. The bed should be so situated as to be easily approached from both sides; and, if convenient, have the head toward the north, to enable the patient to face the sunshine, to look on the out-doors, which has itself a cheering effect, lessens the monotony of the sick chamber, and tends to divert the mind from the condition of the body, thus allowing time to pass more pleasantly.

4. A good ventilation, a constant supply of pure out-door air, is of inestimable advantage to the sick.

5. If a fire is kept on the hearth in cold weather; and in warm, a large lamp is kept burning day and night, and the inner doors always kept open, there is a continual draft passing along the floor towards the fireplace, which carries before it and up the chimney the most deleterious gases in the room, for the heaviest and most poisonous of these are always near the floor.

A good ventilation is obtained by having a board three or four inches wide, and just as long as the window sash, placed edgewise on the sill and under the sash. This causes an opening at the joining of the upper and lower sash, through which a draught of air is driven upward.

6. There should be no standing liquid of any description in a sick-room, not even the purest cold water; because, like a pane of glass in a cold morning, the cold water causes the tainted atmosphere of the sick-room to settle on its surface, and condense into oily drops, which under a microscope exhibit various impurities unfit to be drunk; or the same particles may mingle with the air, be breathed into the lungs and thus be incorporated with the blood.

7. Everything perishable by evaporation should be removed from the room, as food and fruits, because their exhalations contaminate the air.

8. All medicines and bottles, or anything else which reminds of medicine, should be kept out of sight, except at the moment of administering them.

9. There should be no clothing hanging about the room, and as little drapery about the bed and windows as possible, for these hold dust and absorb odors and keep them in the room. For the same reason it is better to have no carpet on the floor, except a strip in front of the bed. In all diseases sleep is better than medicine, and promotes cure more than anything else.

10. The qualifications of the nurse are so varied and so important, that no expense should be spared to secure a good one. Women are generally better than men. The nurse should be a friend or acquaintance with whom the patient has had pleasant associations, for these things secure more devoted attention than hired services. The nurse should be firm and quietly decided; of a kindly, gentle nature, and sympathetic, at the same time active and prompt and should seem cheerful.

11. All short, sharp, sudden movements should be watched against, as they tend to alarm the patient.

12. Mere visitors, if allowed in a sick-room, should remain only long enough for a friendly greeting, with the communication of such intelligence as might make a pleasant impression on the mind. Avoid questioning very sick persons as to the state of their health. They seldom know exactly how they are. Persons recovering from a long and severe illness are usually despondent and think they are going to die just when the physician regards recovery as quite certain.

13. The nurse should not ask the patient if anything is wanted, or what he will have. Nine times out of ten they don't know and the question annoys them. A competent nurse knows best what is needed.

14. Whatever is said to a patient should be as hopeful and assuring as is compatible with truthfulness. Avoid such expressions as "You have changed a great deal," "I would not have known you," "You are very sick," "I am afraid you will not get well," and yet these things have been said to the sick.

15. Never whisper in a sick room, or speak in a low tone. This self-evident propriety is too often disregarded by even rational people. Never look sideways at a patient, or seem to be scanning him; and don't stare at him. The moment you are out of conversation leave the room.

16. Never wake a patient, even to give medicine or to dress a wound, for sleeping is a healing process, giving strength and life. In almost all sickness a tendency to sleep is a favorable sign. Let the patient alone when there is nothing that requires especial attention.

17. The clothing should be changed once a day, and if possible the bed should be made night and morning, and several times a day the bed clothes should be raised up from the body and let fall again, so as to drive out the confined air; or they should be thrown down toward the feet to allow a full airing.

18. Sameness is terrible to one who has been long an invalid; hence, make some changes in the room every day; not many, one or two at a time. Either change the position of the furniture, or introduce a picture or painting, or a bunch of flowers. These may seem to be small matters to the well and hearty, but to the poor invalid, who has been at death's door, they are of incalculable value.

---

The oldest medical work extant is a roll of papyrus obtained by the celebrated German archæologist, Ebers, in Egypt. He was traveling in that country a few years since and learned that a papyrus roll had been discovered lying by the side of a mummy. After considerable difficulty he became possessed of it. It is about eleven inches wide and sixty feet long, and is in excellent preservation. It was written 1522 years before the Christian era, when Moses had just reached his twenty-first year. The author is believed to be the great Thoth, who was deified by the Egyptians on account of the civilization which he brought them. It is the intention of Ebers to make a complete translation of this work.

